# Internet Access Influences Community Clinic Portal Use

**DOI:** 10.1089/heq.2018.0019

**Published:** 2018-08-01

**Authors:** Ruth A. Bush, Halsey Barlow, Alexa Pérez, Bianca Vazquez, Jonathan Mack, Cynthia D. Connelly

**Affiliations:** Hahn School of Nursing and Health Science, Beyster Institute for Nursing Research, University of San Diego, San Diego, California.

**Keywords:** electronic health record, information technology, meaningful use, patient portal, underserved

## Abstract

**Purpose:** To assess whether individuals attending a community clinic had the necessary Internet access and experience to use the patient portal, while examining covariates of education, income, and self-perception of health with past and anticipated portal use.

**Methods:** Adults attending an urban, community primary care clinic were invited to participate in a brief survey assessing current Internet access and use, past portal use, and anticipated future portal use. Survey responses were analyzed using descriptive and multivariate statistics.

**Results:** One hundred fifteen participants ranging in age from 18 to 84 years (mean 42.1, standard deviation 17.1) completed the survey; 6 (5%) in Spanish. Thirty-five (30%) self-identified as Latino; 12 (10%) as Asian; and 20 (17%) as other. Almost 80% reported their health as good or better. Although 38% reported some college and 47% reported being college graduates, 60% reported household incomes were <$50,000. Most (87%) used the Internet for >1 year. Fewer than half (42%) had past portal use, with significant differences associated with weekly Internet use (Fisher's exact=9.59; *p*=0.02) and smart phone access (Fisher's exact=6.15; *p*=0.02). Computer Internet access was significantly associated with income (Fisher's exact=16.91; *p*<0.001). Logistic regression identified that computer Internet access was a significant predictor (odds ratio 9.9 (95% confidence interval: 1.7–58.8) of future portal use, controlling for smart phone use, health status, gender, and age.

**Conclusions:** Among this highly educated but lower economic sample, computer Internet access and smart phone access were associated with past portal use and anticipated future use.

## Introduction

The Health Information Technology for Economic and Clinical Health (HITECH) Act, enacted in 2009, supports the meaningful use of electronic health records (EHRs) to improve patient quality of care.^1,2^ The patient portal is a secure online website providing patients 24 h access to the EHR, designed to facilitate patient engagement, increase self-management, improve patient and health provider communication outside of face-to-face visits, increase patient satisfaction from care received, and result in better clinical outcomes.^3–5^ Patient portals allow individuals to send secure messages to clinical staff, view their health records (e.g., diagnoses, laboratory results, and medications list), schedule appointments, request prescription refills, and manage bills.^6^

Because the portal can be an important tool for scheduling appointments, providing timely communication with providers, and improving understanding of medical conditions and their treatment, it may also serve as an effective engagement tool.^7^ Patient portals have been demonstrated to do the following: improve medication adherence, disease awareness, and self-management of disease, decrease office visits, increase preventative medicine, and increase office visit duration at the patient's request for additional information. The results also show an increase in quality in terms of patient satisfaction and customer retention, but there are weak results on medical outcomes.^8^

Current research demonstrates that portal use is influenced by both health system characteristics and personal factors, such as age, education, and digital literacy.^9^ Diverse patient populations are increasingly using patient portals; in some studies more than half the participants enrolled in the portal.^10,11^ Adult patients who identify as racial and ethnic minorities tend to underutilize portals for a variety of reasons, including a lack of interest in the technology; not feeling that the portal was helpful; physicians not discussing the portal with them; or lack of a computer or Internet access.^10–14^

Several research studies have identified possible barriers to patient portal adoption, including differences in Internet access, computer literacy, and Internet proficiency, often referred to as the digital divide.^15,16^ The digital divide is one explanation for underserved groups being less likely to use portals, with strong correlations to utilization by race and household income.^15–17^ With the increasing emphasis on Health Information Technology (HIT) applications, already disadvantaged groups may be at further risk of poorer health outcomes if they lack access to HIT.^3^

Although smart phones are more affordable and data costs have declined, it is important to evaluate whether these changes are enough to promote patient portal literacy or if technology disparities, especially among patients from lower socioeconomic levels, continue to exist. The aims of this study were to determine and to quantify whether patients attending a community clinic had the necessary Internet access, as well as Internet experience, to be able to use the patient portal following access activation. In addition to Internet access, the study looked at educational level, household income, and self-perception of health as covariates of previous and future portal use.

## Methods

### Setting and participants

This study was conducted in a primarily urban community clinic providing medical care, family planning education, and psychological counseling. Among the overall clinic population, ∼65% of patients rely on government-supported insurance; 3% report no insurance coverage. Forty-five percent of patients self-report as Latino and 25% Asian/Pacific Islander. The clinic adopted the eClinicalWorks EHR in August 2013, which includes a patient portal with both an English and a Spanish interface. The portal can be accessed using an online webpage and on a smart phone application following a two-stage activation, in which individuals can log into their account after receiving an initial system email with temporary login information generated at the individual's request. At the time of the study, the clinic had 14340 registered individuals of whom 1511 (10.5%) were portal enabled.

### Survey

We adapted and translated into Spanish the survey designed by Taha et al.^18^ to document portal usability barriers into a one-page, two-sided survey with English on one side and Spanish on the other. From March 2017 to October 2017, a bilingual, bicultural research associate recruited potential participants from a convenience sample as they waited for a regularly scheduled clinic visit. The survey took 3–5 min to complete and consisted of demographics; self-reported health rating; type of Internet access (phone, tablet, and computer); length of Internet experience; current weekly Internet use; past portal use; and anticipated future portal use. (Complete survey is presented in Supplementary Appendix A1) Participants were eligible if they were receiving care from the clinic, 18 years or older, English or Spanish readers, and willing to complete the survey. All survey refusals were logged and a brief reason for refusal documented. No remuneration was provided. All study procedures were approved by the appropriate Institutional Review Board and clinic administrators for the protection of human subjects.

### Data sources and analysis

Survey data were assessed to identify if technology access or patient preferences served as barriers to adolescent portal use. SPSS (version 23)^19^ was used for analysis. Data reports generated from the EHR were also analyzed as a secondary measure to examine the overall portal signup and activation. Descriptive analyses were performed to describe demographic variables, technology use, and access. After determining bivariate significance using independent *t* tests and Fisher's exact tests, a saturated logistic regression model was created to evaluate the effect of socioeconomic factors on technology. With >20 cases for each independent variable included in the logistic regression model, there was sufficient power for the analysis.

## Results

The research staff approached 254 individuals during clinic appointments during the study period. Of those approached, 115 agreed to complete a survey (45.3%). Reasons for not participating included no perceived need for portal; no time that day; not feeling well; concern about the security of the portal; reluctance to share personal information (although the survey was deidentified); and no reason given.

### Participants demographics

Selected demographics for survey participants are found in Table 1 (*N*=115). One hundred fifteen participants ranging in age from 18 to 84 years (mean 42.1) completed the survey. Six participants (5%) completed the survey in Spanish. Thirty-five (30%) of the participants self-identified as Latinos; 12 (10%) as Asians; and 20 (17%) as other. Almost 80% reported their health as good or better. Although 38% reported completing some college and 47% reported being college graduates, 60% reported a household income of <$50,000.

**Table 1.** **Selected Demographics from Participants Who Completed the Portal Survey**

Demographic	*n* (%)
Age (SD)	42.1 (17.1)
Gender	
Male	27 (23.5)
Female	88 (76.5)
Race/ethnicity	
Latino/Hispanic	35 (30.4)
Asian	12 (10.4)
White	48 (41.7)
Other	20 (17.4)
Education	
High school	17(14.8)
Some college	43 (37.4)
College graduate	53 (46.1)
Income	
<$20,000	27 (23.5)
$20,000–49,000	39 (33.9)
$50,000 or more	45 (39.1)
Self-reported health	
Poor	3 (2.6)
Fair	22 (19.1)
Good	55 (47.8)
Very good	26 (22.6)
Excellent	8 (7.0)

SD, standard deviation.

### Survey results

Ninety participants responded either having access to a smart phone or to a computer with Internet access, indicating that there was not a significant “digital divide” among this community clinic population. However, Table 2 shows that there were individuals with limited or nonexistent access. There were few responses for tablet use, and the question was dropped from the analysis. Within the sample, 87% had used the Internet for >1 year and 60% reported using the Internet for 5 or more hours a week. Forty-seven (42%) had used the portal in the past. There were significant differences in past portal use by reported time spent on the Internet during the week (Fisher's exact=9.71; *p*=0.02) and smart phone Internet access (Fisher's exact=6.15; *p*=0.02). Using a computer for Internet access was significantly associated with income level (Fisher's exact=16.91; *p*<0.001).

**Table 2.** **Participant Characteristics and Past Portal Use**

	Past portal use			
	Yes	No	Pearson's chi-square	Fisher's exact *p*-value
Age				
18–34	20	28	5.68	0.06
35–64	23	22		
65 and older	3	14		
Gender				
Female	40	47	2.18	0.17
Male	7	17		
Race/ethnicity				
Non-Latino/Hispanic White	20	27	0.31	0.96
Latino/Hispanic	14	20		
Other	8	12		
Asian	5	5		
Education				
High school graduate	4	13	4.34	0.11
Some college	16	26		
College graduate	26	25		
Income				
<$20,000	9	17	4.07	0.13
$20,000–$49,000	13	24		
$50,000 or more	24	20		
Health status				
Poor to fair	12	12	1.07	0.59
Good	22	31		
Very good to excellent	12	21		
Internet experience Internet use (weekly)				
<1 year	4	10	1.29	0.39
Year or more	42	52		
<1 h	3	12	9.71	**0.02**
1–5 h	8	20		
>5 h and <10 h	16	12		
10 h or more	20	17		
Smart phone				
Yes	44	47	6.15	**0.02**
No	3	15		
Computer				
Yes	40	51	0.33	0.62
No	7	12		

Bold values are statistically significant.

There was no significant age- or gender-based difference in basic technological access. Seventy-eight percent of respondents indicated that they would like to use the portal in the future to allow access to their EHR (Table 3). There were significant differences between age and gender regarding their desire to use a patient portal with women significantly more interested in future portal use (Fisher's exact=8.61; *p*=0.006) and those younger than the age of 65 years or more likely to indicate interest in future portal use (Fisher's exact=11.59; *p*=0.003).

**Table 3.** **Participant Characteristics and Anticipated Future Portal Use**

	Future portal use			
	Yes	No	Pearson's chi-square	Fisher's exact *p*-value
Age				
18–34	44	4	11.59	**0.01**
35–64	37	8		
65 and older	8	7		
Gender				
Female	75	10	8.61	**0.01**
Male	15	9		
Race/ethnicity				
Non-Latino/Hispanic White	40	7	1.81	0.69
Latino/Hispanic	25	8		
Other	15	3		
Asian	10	1		
Education				
High school graduate	12	5	2.89	0.24
Some college	32	7		
College graduate	45	6		
Income				
<$20,000	22	5	1.27	0.53
$20,000–$49,000	29	8		
$50,000 or more	36	5		
Health status				
Poor to fair	16	7	5.30	0.08
Good	44	10		
Very good to excellent	29	2		
Internet experience internet use (weekly)				
<1 year	10	5	3.40	0.13
Year or more	79	13		
<1 h	11	4	5.60	0.13
1–5 h	19	8		
>5 h and <10 h	25	3		
10 h or more	33	4		
Smart phone				
Yes	80	11	8.73	**0.01**
No	10	7		
Computer				
Yes	77	12	5.25	**0.03**
No	13	7		

Bold values are statistically significant.

**FIG. 1.**
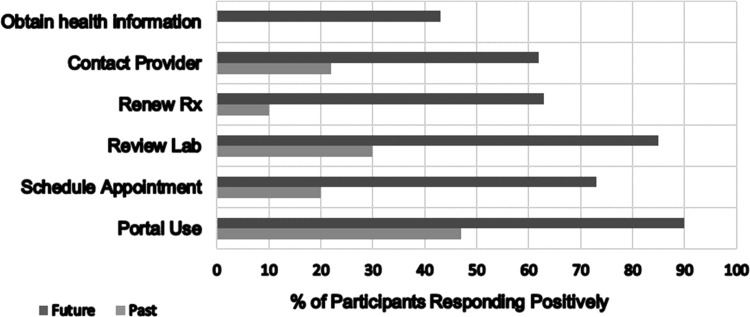
Portal function past use and anticipated future use.

Figure 1 illustrates the ways in which individuals used the portal in the past and how they anticipate using it in the future. Reported past use varied from looking at test results (31%); communicating securely with providers (20%); scheduling appointments (18%); and requesting prescription refills (9%). Ninety respondents (83%) would like to use the portal in the future, primarily to view test results (79%), for secure communication with provider (59%), and prescription refills (59%).

Logistic regression was used to determine the relationship of the predictors significant in the bivariate analysis to anticipated future portal use, including smart phone Internet access, computer access, self-reported health status, sex, and age. With an overall chi-square of 30.34 (*p*=0.001), a Nagelkerke R-squared of 0.42; and a classification of 89.4%, computer Internet access (odds ratio [OR] 9.9, 95% confidence interval [CI]: 1.71–58.82) was the only significant variable in the model when controlling for the other independent variables. Gender was trending, with females more likely than men to indicate anticipated future use (OR 4.2, 95% CI: 1.00–17.58).

## Discussion

Among this highly educated but lower income sample, there was a high but not universal level of access to the Internet as well as weekly use of the Internet, which has been previously demonstrated.^20^ In addition, this study explored assessed Internet gateway modality with past portal use and anticipated future use, which highlights obstacles to overcome as well as strategies for future portal engagement.

Overall, our results indicate that many but not all individuals attending a community clinic have the needed Internet access to use the patient portal. While there was not a profound “digital divide” among this primarily urban, low socioeconomic status population, not all report the basic technology needed to access the patient portal. As evidence of this, 17 (15%) of the 115 individuals reported neither smart phone nor computer access needed for portal use. The disparity in technology access and inability to adopt a growing technological approach to clinical practice puts these individuals at increased risk for poor clinical outcomes.^3^

The survey data demonstrated that individuals with varying education level and self-reported health status have an active interest in using patient portals, although interest waned among those older than 65 years, unlike another recent study in a similar sample.^21^ Surveyed individuals expressed an interest in future use of email or text messaging as a means of communicating with their providers, indicating that they are willing to consider use of electronic communication with their healthcare provider. It is notable that ∼10% of individuals registered with this clinic actually do use the portal. These findings are similar to other studies that found that perceived need of a portal does not match actual usage.^22,23^ Although HIT and communication technology continue to play an integral role in proposed solutions for patient engagement (including individuals from lower-socioeconomic levels) by providing a critical link among patients, needed health resources, and available service, the technology is not being utilized at desired and impactful levels.^4,15–17,24^

Even though there was no difference in past or anticipated future use of portal by income, the significance of using the computer, which was statistically associated in this sample with higher income, to access the portal may reflect the advantage of less restricted Internet access without data allowances and the need to be able to interact with the relatively complex medical record on a screen that is large enough to see the information in a single view without scrolling. This finding is consistent with another large, urban, heterogeneous study, in which mobile phone and tablet use of the portal was limited compared with the desktop usage and negatively impacted activation rates among those without regular computer access,^25^ which reinforces the existence of potential portal barriers such as slow or nonexistent Internet access, resistance or little time for data entry, need for complex language comprehension, intricate visual layouts, and poor usability features.^26–29^

Our findings must be considered in light of several general limitations. The survey was administered to a convenience sample, did not capture all possible participants, and had a 45% participation rate. The diverse race/ethnicity profile of the participation undersampled the clinic's Latino/Hispanic population. In addition, while our participants were mainly low-income people, they were fairly highly educated adults attending a community clinic, which may reduce the generalizability of our findings. This study would be further strengthened if the participants had also completed measures of health literacy and technologic expertise as reported technology use does not equate to technologic proficiency.

## Conclusion

Among this highly educated but lower economic sample, there was a high level of access to the Internet, as well as weekly use of the Internet, but such access and use was not yet universal. Within the sample, use and technological gateway modality such as using a smart phone and having a computer were not only associated with past use but also the likelihood of future use. Although there was no difference in past or anticipated future use of portal by income, the significance of using the computer, which was associated with higher income in this sample, may indicate better Internet access and the ability to more easily interact with the portal on a larger screen. As many lower income individuals only have Internet access via a smart phone, this is an important finding when considering disparities in potential patient engagement.

## Supplementary Material

Supplemental data

## Abbreviations Used

CI: confidence interval
EHR: electronic health record
HIT: Health Information Technology
OR: odds ratio
SD: standard deviation
