# Supplementary material for: Internet Access Influences Community Clinic Portal Use
Source: Health Equity. 2018 Aug 1;2(1):161–6. doi: 10.1089/heq.2018.0019 (PMC6110185; doi:10.1089/heq.2018.0019)
Supplement: Supplemental data [file Supp_Data.pdf]

SUPPLEMENTARY DATA

Supplementary Appendix A1

Patient Portal Survey

As a patient of the Clinic Culture of Family and Life Services, we are asking for your help. We have been testing our clinic patient portal, as a way to help you securely and confidentially communicate with your doctor's office. Our clinic Patient Portal can be securely accessed online from your home or office, or by using your mobile smartphone. In the future, it will be used to email your doctor, view your test results, schedule appointments, and renew your prescription. We would value your feedback on ways in which you think we can make the clinics patient portal better. This survey takes about 5 minutes to complete. Your participation in this survey is completely voluntary, anonymous, and your answers are confidential; it will not affect your health service.

1. Age ______________________
2. Gender (Please Choose One):
   - ☐ Male
   - ☐ Female
   - ☐ Other
3. Ethnicity (Please Choose One):
   - ☐ Latino/Hispanic
   - ☐ Non-Latino/Hispanic White
   - ☐ Non-Latino/Hispanic Black
   - ☐ Asian
   - ☐ Native American
   - ☐ Other: ______________________
4. Education:
   - ☐ Less than High School Graduate
   - ☐ High School Graduate
   - ☐ Some College
   - ☐ College Graduate
   - ☐ Graduate School
5. Household Income:
   - ☐ < $20,000
   - ☐ $20,000 to $49,999
   - ☐ $50,000 or more
6. Ratings of General Health:
   - ☐ Poor
   - ☐ Fair
   - ☐ Good
   - ☐ Very Good
   - ☐ Excellent
7. Length of time using the Internet:
   - ☐ Less than 6 months
   - ☐ Between 6 months and a year
   - ☐ More than 1 year, but less than 5 years
   - ☐ 5 years or more
8. Hours/week using the Internet:
   - ☐ Less than 1 hour
   - ☐ Between 1 and 5 hours
   - ☐ More than 5 hours, but less than 10 hours
   - ☐ 10 hours or more
9. How do you access the Internet? (Check all that apply):
   - ☐ Phone
   - ☐ Tablet
   - ☐ Computer
10. Have you used a Patient Portal in the past?
    - ☐ Yes
    - ☐ No
11. How did you use the Portal? (Check all that apply):
    - ☐ Schedule appointments
    - ☐ View test results
    - ☐ Renew prescriptions
    - ☐ Send and receive emails from your health-care providers
    - ☐ Link to health information from trustworthy sources on the Internet
12. Would you like to use the Portal in this clinic?
    - ☐ Yes
    - ☐ No
13. How would you use the Portal? (Check all that apply):
    - ☐ Schedule appointments
    - ☐ View test results
    - ☐ Renew prescriptions
    - ☐ Send and receive emails from your health-care providers
    - ☐ Link to health information from trustworthy sources on the Internet

Cuestionario del Portal de Pacientes

Como paciente de esta Clínica de La Cultura de la Familia y Servicios de la Vida (COLFS, Culture of Life Family Services) le estamos pidiendo su ayuda. Hemos estado probando nuestro portal de pacientes aquí en la clínica, como una manera de ayudarle a comunicarse con la oficina de su proveedor de salud, de una forma más segura y confiable. Nuestro Portal de Pacientes de la clínica se puede acceder en línea

de una manera segura desde su casa u oficina, o utilizando su teléfono celular. En el futuro será utilizado para enviarle correos electrónicos a su médico, ver sus resultados de laboratorio, programar citas y renovar recetas médicas. Nosotros valoramos su opinión acerca de las maneras en las que usted cree que podemos mejorar el portal de pacientes en nuestra clínica. Este cuestionario le tomara aproximadamente 5 minutos para completar. Su participación en este cuestionario es completamente voluntario, anónimo y sus respuestas son confidenciales y no afectará de ninguna manera a sus servicios de salud.

1. Edad: ________________________
2. Sexo (Por favor Seleccione uno):
   - ☐ Masculino
   - ☐ Femenino
   - ☐ Otro
3. Etnicidad (Por favor seleccione uno):
   - ☐ Latino/Hispano
   - ☐ No-Latino/Hispano Blanco
   - ☐ No-Latino/Hispano Negro
   - ☐ Asiático
   - ☐ Nativo Americano
   - ☐ Otro: ________________________
4. Educación:
   - ☐ Menos del grado 12º
   - ☐ Grado 12º ó Equivalente (GED)
   - ☐ Algo de Universidad
   - ☐ Titulo universitario o Licenciatura
   - ☐ Titulo Postgrado
5. Ingresos Anuales de la familia:
   - ☐ < $20,000
   - ☐ $20,000 a $49,999
   - ☐ $50,000 o más
6. Valoración de la Salud en general:
   - ☐ Pobre
   - ☐ Medio
   - ☐ Bueno
   - ☐ Muy Bueno
   - ☐ Excelente
7. Tiempo que lleva utilizando el internet:
   - ☐ Menos de 6 meses
   - ☐ Entre 6 meses y 1 año
   - ☐ Más de 1 año, pero menos de 5 años
   - ☐ 5 años o más
8. Horas/semana que utiliza el Internet:
   - ☐ Menos de 1 hora
   - ☐ Entre 1 y 5 horas
   - ☐ Más de 5 horas, pero menos de 10 horas
   - ☐ 10 horas o más
9. ¿Cómo accede a internet? Seleccione todas las que apliquen:
   - ☐ Teléfono
   - ☐ Tableta Electrónica
   - ☐ Computadora
10. ¿Ha utilizado un Portal de Pacientes en el pasado?
    - ☐ Si
    - ☐ No
11. ¿Como utilizó el Portal de Pacientes? (Seleccione todas las que aplique):
    - ☐ Programar Citas
    - ☐ Ver resultados de laboratorio
    - ☐ Renovar recetas medicas
    - ☐ Mandar y recibir correos electrónicos de sus proveedores de salud
    - ☐ Acceder a información medica de fuentes confiables en internet
12. ¿Le gustaría utilizar el Portal de Pacientes de esta clínica?
    - ☐ Si
    - ☐ No
13. ¿Cómo utilizaría el Portal de Pacientes?(Seleccione todas las que apliquen):
    - ☐ Programar Citas
    - ☐ Ver resultados de laboratorio
    - ☐ Renovar recetas medicas
    - ☐ Mandar y recibir correos electrónicos de sus proveedores de salud
    - ☐ Acceder a información médica de fuentes confiables en internet
